# Prevalence and Risk Factors of Modern-Type Depression in Oman: Validation of the Arabic TACS-22 Scale

**DOI:** 10.1192/j.eurpsy.2025.1298

**Published:** 2025-08-26

**Authors:** S. Al Busaidi, S. Al Adawi, M. F. Chan

**Affiliations:** 1 psychiatry, Resident doctor at Oman medical speciality board; 2 Department of Behavioral Medicine; 3 Department of Family Medicine & Public Health, College of Medicine & Health Sciences, Sultan Qaboos University, muscat, Oman

## Abstract

**Introduction:**

Emerging mental health crises in the global south highlight challenges in applying psychological frameworks that primarily originate from wealthy nations in the global north. The concept of modern-type depression (MTD), first recognized in Japan, has recently gained attention due to its divergence from the DSM criteria. MTD manifests itself as a reluctance to engage in social roles, a withdrawal from social norms, and vague omnipotence. Oman, a rapidly developing nation, shows patterns of social withdrawal similar to MTD. This study aims to validate the Arabic version of Tarumi’s Modern Depression Trait Scale (TACS-22), evaluate its factor structure, and explore the prevalence and risk factors of MTD in Oman.

**Objectives:**

The entrance of urbanization and digitation has heralded ‘de novo psychopathologies’ such as those that go under the umbrella of modern-type depression (MTD). To date, the presence of the MTD and the factors associated with it have yet to be explored. Related aims are to conduct exploratory factor analysis (EFA) and confirmatory factor analysis (CFA) to confirm the factorial validity of the 22-item Tarumi’s Modern-Type Depression Trait Scale.

**Methods:**

An online survey was conducted in March 2023 with a convenience sample of Omanis aged 18 and over. The survey included the Arabic-translated TACS-22 scale and the PHQ-9 to assess depressive symptoms. An exploratory factor analysis (EFA) was performed to determine the structure of the TACS-22 factor and internal consistency was assessed using Cronbach’s alpha. Sociodemographic and clinical data was collected to explore possible risk factors for MTD and univariate and multivariate logistic regression analyses were performed to examine associations.

**Results:**

A total of 1009 participants completed the survey, with a response rate of 50%. The majority were female (70.3%) with a mean age of 31.1 ± 8.5 years. The prevalence of MTD was 25.3%, with significant associations found between MTD and sociodemographic factors such as sex, marital status, age, and occupation (p < 0.05). Furthermore, clinical factors such as mental illness, adverse childhood experiences (ACE), and depressive symptoms were also significantly associated with MTD. The EFA revealed a three-factor structure: Avoidance of social roles, low self-esteem, and complaints, consistent with previous studies, with acceptable internal consistency (Cronbach’s α = 0.77).

**Conclusions:**

MTD is prevalent in Oman, particularly among younger individuals, students, and the unemployed. The Arabic TACS-22 scale demonstrates acceptable validity and reliability, providing a useful tool for identifying MTD traits in non-Western contexts. Socioeconomic changes in Oman can contribute to the emergence of MTD, and further research is needed to explore tailored interventions for this population.

**Disclosure of Interest:**

None Declared

